# Effects of BCI-based lower limb robots on lower limb function and cognition in stroke patients: a preliminary systematic review

**DOI:** 10.3389/fnhum.2026.1782862

**Published:** 2026-07-02

**Authors:** Mengqi Shao, Xiurong Wang, Kui Wang, Kang Yang, Andi Zou, Bo Chen, Hui Zhang, Zhaoxiang Meng, Xing Jin

**Affiliations:** 1 Rehabilitation Medicine Department, Yangzhou Second People’s Hospital, Yangzhou, Jiangsu, China; 2 Blood Purification Center, Northern Jiangsu People’s Hospital, Yangzhou, Jiangsu, China; 3 Rehabilitation Medicine Department, Northern Jiangsu People’s Hospital, Yangzhou, Jiangsu, China; 4 Rehabilitation Medicine Department, Sunshine Union Hospital, Weifang, Shandong, China

**Keywords:** BCI, cognition, lower limb, robotics, stroke, systematic review

## Abstract

**Background:**

BCI-based lower limb robots (BCI-LLR) represent a novel technology used in neurological rehabilitation for stroke patients. However, the effectiveness of BCI-LLR compared to traditional rehabilitation in improving lower extremity function and cognition remains a topic of debate. This study aimed to determine whether BCI-based lower limb robots are more effective than traditional rehabilitation for lower limb dysfunction after stroke.

**Methods:**

A comprehensive search was conducted across multiple databases, including PubMed, Embase, Web of Science, and Cochrane Library. The database retrieval was performed up until November 21, 2025. A systematic review of findings was conducted due to the limited number and the heterogeneity of studies.

**Results:**

A total of four studies (three RCTs and one single-arm pre-post study) with 117 stroke patients were included. BCI-LLR training significantly improved lower limb function, with one RCT showing significant between-group differences in FMA-L (+4.5 vs. +2.1, *p* = 0.022), and another RCT demonstrating significant advantages in TUG (−12.03 vs. −4.65 s, *p* = 0.038) and BBS (+5.50 vs. +3.38, *p* = 0.042). The single-arm study also reported significant improvements in all lower limb outcomes (*p* < 0.01). For cognition, SDMT improvements significantly favored BCI-LLR in two RCTs (*p* = 0.036 and *p* = 0.013) and in the single-arm study (*p* = 0.047). MoCA showed improvement only in the single-arm study (*p* = 0.044). The attention index increased significantly in three studies (*p* < 0.01). Neurophysiological benefits were reported in one RCT (*p* < 0.05).

**Conclusion:**

Preliminary evidence suggests BCI-LLR may improve lower limb function (FMA, BBS, TUG) and information processing speed (SDMT). However, effects on global cognition (MoCA) remain unclear, and sample sizes are critically small.

**Systematic review registration:**

This study was registered on the international system evaluation registration platform PROSPERO (CRD420251232191).

## Introduction

Stroke is a neurological disease caused by the rupture or blockage of a cerebral blood vessel. It triggers a cascade of pathophysiological events, including excitotoxicity, oxidative stress, and neuroinflammation, ultimately leading to neuronal death (Purrahman et al., 2023). Stroke is the second leading cause of disability and death worldwide and remains one of the most disabling neurological conditions (Saini et al., 2021). Survivors often experience significant declines in mobility and dynamic balance (Żukowska et al., 2025), placing a substantial burden on society (Momtazmanesh et al., 1990).

After a stroke, most patients continue to have limb motor dysfunction despite months or years of rehabilitation. Approximately 70% of patients experience slowed gait or hemiplegia (Li et al., 2021). Among them, lower limb dysfunction is a common disorder of functional loss. The pathophysiological mechanism of motor dysfunction after stroke is complex. The underlying mechanism is that nerve damage disrupts the neural substrate for motor control and coordination (Le Franc et al., 2022), which directly affects the strength and coordination of lower limb muscles. Due to this gait pattern, stroke patients lack independence, which not only limits the patient’s ability to walk, but also significantly increases the risk of falling due to impaired balance (Khalid et al., 2023). This limits patients’ daily activities and reduces their quality of life (Elameer et al., 2023; Hornby et al., 2020). Therefore, restoring safe and independent walking is a primary goal for stroke survivors. This is an important milestone in rehabilitation for stroke survivors to regain the ability to walk.

A stroke leaves the affected limb with little or no function. Patients generally move their paralyzed limbs through imaginary exercises, but it is often difficult to perform high-intensity exercises in traditional rehabilitation. Lower limb rehabilitation robots provide the sensory input needed to improve walking ability, which can effectively maintain the patient’s joint range of motion and gradually enhance the patient’s gait during rehabilitation training (Li et al., 2024). In recent years, new types of assisted rehabilitation robots that transcend traditional designs have also emerged. As a cutting-edge technology integrated by multiple disciplines, brain-computer interfaces are gradually receiving attention and application. They can use brain signals in recorded neural responses to generate various control signals or instructions, allowing individuals to directly control external devices using brain signals, without relying on speech or body movements, to interact with the external environment (Hossain et al., 2023).

Previous meta-analyses have shown that conventional lower-limb rehabilitation robots (without BCI) can improve the Berg Balance Scale and Fugl-Meyer lower limb scores more effectively than conventional rehabilitation alone. Ordinary lower limb rehabilitation robots (without BCI) can effectively improve the walking function, balance ability and daily living activities of hemiplegic patients (Hu et al., 2024; Hao et al., 2025). However, brain-computer interface lower limb rehabilitation robots (BCI-LLR) are hypothesized to provide additional benefits by integrating motor imagery and real-time neurofeedback, potentially enhancing neuroplasticity more than ordinary robots.

BCI-LLR reports visual, auditory, or tactile feedback to promote neuroplasticity and motor regeneration in patients by capturing electroencephalograms during patient training (Baniqued et al., 2021). Compared with ordinary lower-limb rehabilitation robots, BCI-LLR continuously monitors patient intentions to adjust exercise intensity and patterns in real time, providing personalized and adaptive rehabilitation, thereby improving training results (Esposito et al., 2021). Based on the idea of plasticity of the central nervous system, BCI-LLR completes multi-functional, physiological simulated exercises through multiple parameter settings. This personalized and interactive rehabilitation training method can not only increase the patient’s sense of participation (Liu et al., 2023), but is also an efficient and safe form of rehabilitation treatment.

At present, upper limb robots based on BCI have been confirmed to have good curative effects, compared with ordinary robots. However, only a few studies have comprehensively investigated the effects of conventional lower limb robots, and the efficacy of BCI-based lower limb robots (BCI-LLR) remains unconfirmed. Therefore, this article aims to discuss whether BCI-LLR has better effects on lower limb dysfunction after stroke. In addition, the BCI-LLR innovatively incorporates intention adjustment, so we also included a discussion on whether it can improve cognition.

## Method

The Preferred Reporting Items for Systematic Review and Meta-Analyses (PRISMA) guidelines were followed for the methodology of this study. This study was registered on the international system evaluation registration platform PROSPERO (CRD420251232191).

### Search strategy

We comprehensively obtained relevant literature by searching the PubMed, Embase, Web of Science, and Cochrane Library databases. A combination of subject words and free words was used for the search. He search was conducted from database inception to November 21, 2025. The search terms used include “stroke,” “brain-computer interface,” “lower limbs” and their free words.

The following search strategy was used for PubMed (adapted for other databases):

(“Stroke”[Mesh] OR “stroke”[tiab] OR “cerebrovascular accident”[tiab] OR “CVA”[tiab] OR “hemiplegia”[Mesh] OR “hemiplegia”[tiab] OR “hemiparesis”[tiab]) AND (“Brain-Computer Interfaces”[Mesh] OR “brain-computer interface”[tiab] OR “BCI”[tiab] OR “brain computer interface”[tiab] OR “motor imagery”[tiab] OR “MI”[tiab]) AND (“Lower Extremity”[Mesh] OR “lower limb”[tiab] OR “leg”[tiab] OR “gait”[Mesh] OR “gait”[tiab] OR “walking”[tiab]) AND (“randomized controlled trial”[Publication Type] OR “Randomized Controlled Trials as Topic”[Mesh] OR “RCT”[tiab] OR “randomized controlled trial”[tiab]).

### Inclusion criteria and exclusion criteria

#### Inclusion criteria

1. The study designs included RCTs, non-randomized controlled trials, and single-arm pre-post studies that examined BCI-based lower limb robots;
2. Patients who have acquired lower limb dysfunction after stroke.
3. The experimental group received lower limb rehabilitation exercises based on BCI.
4. Outcome indicators include one or more of Fugl-Meyer Assessment (FMA), Berg Balance Scale (BBS), Timed Up and Go Test (TUG), Symbol Digit Modalities Test (SDMT), and Montreal Cognitive Assessment (MoCA).

#### Exclusion criteria

1. Abstracts, reviews, case reports, or meta-analysis.
2. Lack of data on outcome metrics;
3. Articles with too low research quality and retracted articles.

### Data collection and quality assessment

The included studies were independently assessed by 2 researchers. First, a preliminary screening of titles and abstracts was conducted, the full texts of the screened articles were reviewed, and whether the studies should be included was determined in strict accordance with the inclusion criteria. For RCTs, the Cochrane Risk of Bias tool (RoB 2.0) was used. For the single-arm pre-post study, the JBI Critical Appraisal Checklist for Case Series was applied. The researchers carefully read each study and determined whether the study had a high risk of bias, low risk of bias, or unclear risk of bias. If the opinions of the 2 researchers were inconsistent, the third researcher reviewed the data and a decision was made through discussion. Due to the nature of BCI-LLR interventions, blinding of participants and personnel was rarely feasible, leaving a significant proportion of the included studies at high risk of performance bias. This is an inherent limitation of this type of rehabilitation research. This analysis included extraction of the following data: basic information (ie, author, year of publication, country of publication), experimental data (eg, patient age, number of experimental groups, number of control groups, intervention measures, intervention time), and outcome indicators.

### Statistical analyses

Data synthesis was performed narratively. Individual study results were tabulated and described. No meta-analysis was conducted because of the small number and clinical/methodological diversity of the included studies.

## Results

### Search results

**Figure 1:**
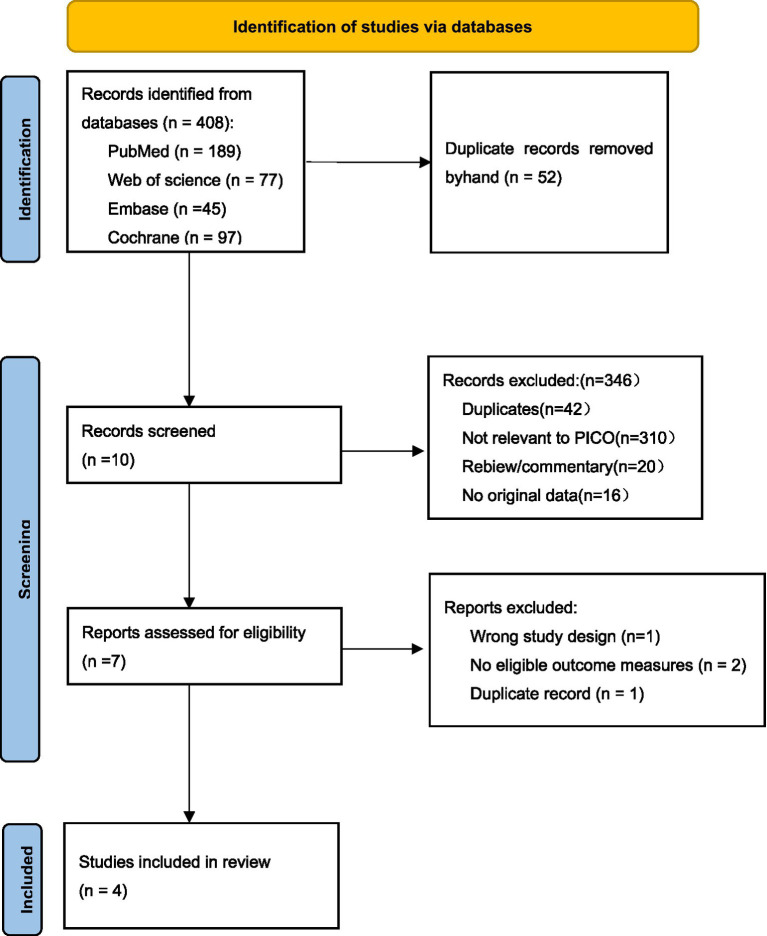
PRISMA flow diagram of the study selection process.

**Figure 2:**
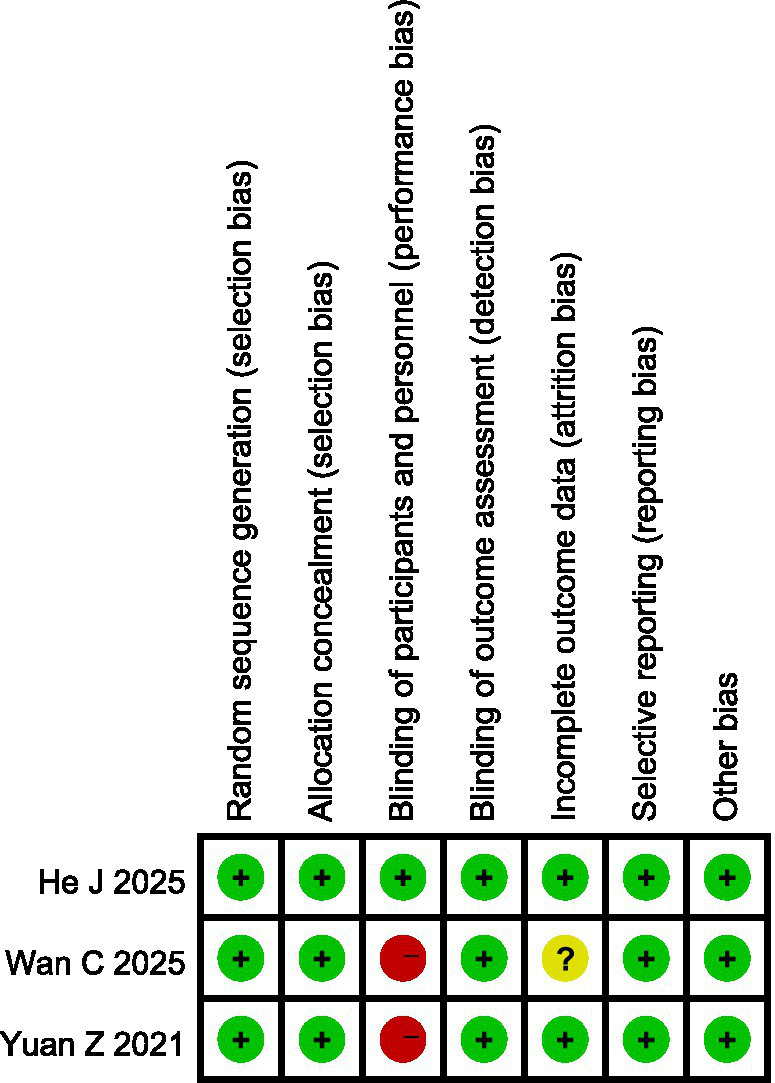
Risk of bias summary: review authors’ judgements about each risk of bias item for each included study.

**Figure 3:**
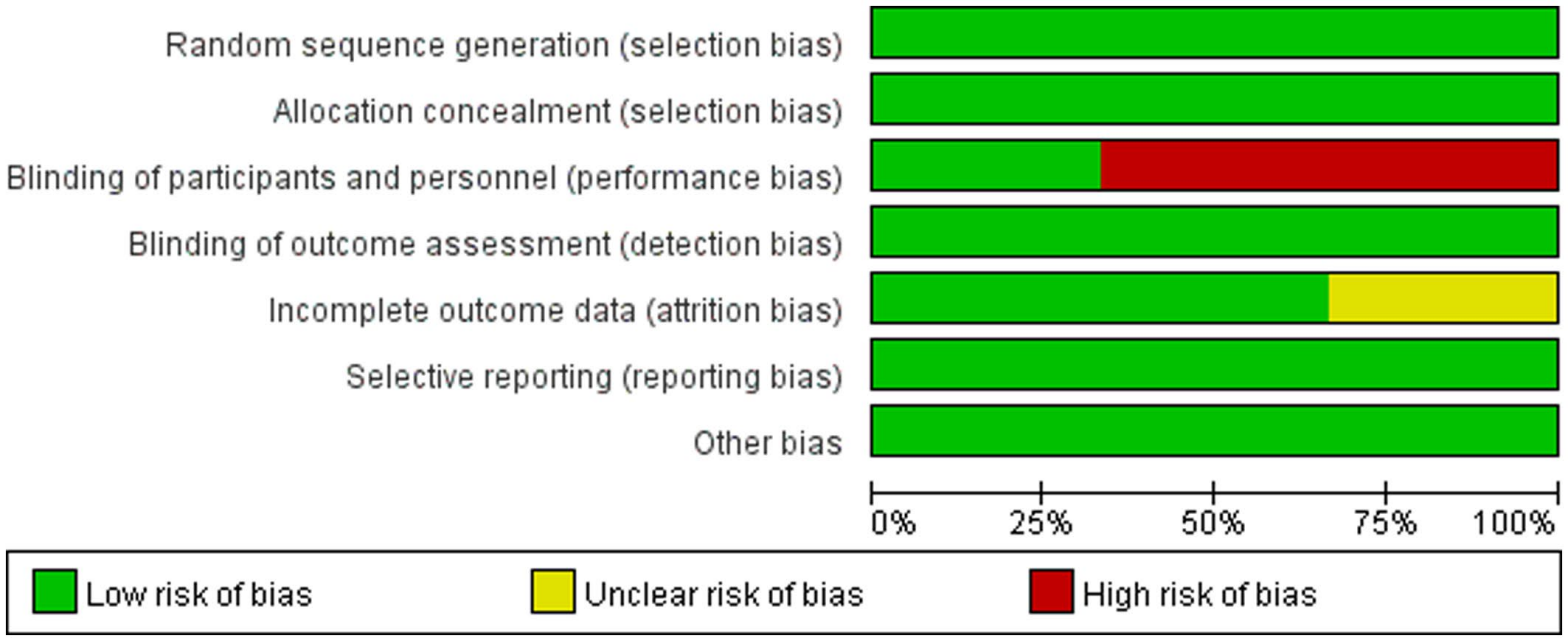
Risk of bias graph: review authors’ judgements about each risk of bias item presented as percentages across all included studies.

A total of 189 articles were retrieved, and 10 articles remained after preliminary screening. After reviewing the full texts, we retained the articles that reported the required outcome indicators, finally obtaining 4 articles. Figure 1 presents the PRISMA flow diagram of study selection. The included studies comprised 3 RCTs and one single-arm pre-post study. The RCTs were assessed with the Cochrane RoB 2 tool, showing some concerns mainly in performance bias; the pre-post study (Wan et al., 2025a) was evaluated with the JBI checklist and had several methodological limitations. Among the four included studies, three had a high risk of performance bias due to the impossibility of blinding participants and personnel, while one study (He et al., 2025) successfully implemented blinding using a sham feedback control and had a low risk of performance bias. Overall, the included studies were of moderate quality, but the high risk of performance bias is an inherent limitation of this type of research. The risk of bias assessment for the three RCTs is presented in Figures 2, 3. Overall, two RCTs had some concerns due to lack of blinding, while one RCT (He et al., 2025) was rated as low risk. The JBI case series critical assessment checklist used in single-arm studies is shown in Table 1.

**Table 1:** JBI critical appraisal checklist for case series.

Criteria	Rating	Explanation
1. Were there clear criteria for inclusion in the case series?	Yes	Inclusion/exclusion criteria explicitly stated (Table 2).
2. Was the condition measured in a standard, reliable way for all participants?	Yes	Standardized, validated scales used (FMA-LE, TUG, BBS, MoCA, SDMT).
3. Were valid methods used for identification of the condition?	Yes	Stroke confirmed by clinical and imaging criteria.
4. Did the case series have consecutive inclusion of participants?	Unclear	Not explicitly stated.
5. Did the case series have complete inclusion of participants?	Yes	12 enrolled, 9 completed (75%); reasons for dropout reported.
6. Was there clear reporting of the demographics of the participants?	Yes	Age, sex, stroke type, lesion location reported (Table 1).
7. Was there clear reporting of clinical information of the participants?	Yes	Baseline FMA-LE, TUG, BBS, MoCA, SDMT reported.
8. Were the outcomes or follow-up results clearly reported?	Yes	Pre-post data with means, SDs, t-values, *p*-values.
9. Was there clear reporting of the presenting site(s)/clinic(s) demographic information?	Yes	Single specialized rehabilitation hospital described.
10. Was statistical analysis appropriate?	Yes	Paired *t*-tests/Wilcoxon tests used appropriately.

Total score: 9/10 (item 4 = unclear) overall quality: high.

### Description of included studies

All the included literature came from China. The study subjects were adult patients who had difficulty in moving their lower limbs after stroke. A total of 117 patients were included, including 76 males and 41 females. The total sample size (*N* = 117) is extremely small and limits the generalizability of findings across studies. All intervention groups received BCI-LLR training, but with different protocols: Yuan used a BCI-controlled pedaling system; Wan et al. (2025a,b) combined BCI-LLR with virtual reality (VR) training; He employed a BCI system integrating motor imagery and motor attempts with a pedaling robot for both upper and lower limbs. Control groups (in the RCTs) received conventional rehabilitation programs without BCI feedback. In these literatures, Wan’s two studies intervened for 4 weeks and articles of Yuan and He intervened for 2 weeks. Detailed characteristics are presented in Table 2.

**Table 2:** Characteristics of included studies.

Authors	Year	Inclusion criteria	Exclusion criteria	intervention	Sample (E/C)	Outcome measures	Period
Yuan Z	2021	1. Aged 40–80 years old; 2. First subcortical ischemic stroke onset from 1 week to 3 months; 3. Hemiplegia and cognitive impairment (MMSE <28 or MoCA <25); 4. Conscious; 5. Sitting balance level 1 or above; 6. Can cooperate with assessment and treatment; 7. Signed informed consent	1. Severely impaired cognition or inability to pay attention to and understand screen information; 2. Severe lower extremity pain or spasticity preventing pedaling training	BCI-LLR	16/14	FMA-LE, SDMT, MoCA	2 W
Wan C	2025	1. Age 18–75 years; 2. Stroke onset within 1–12 months; 3. Lower-extremity Brunnstrom stage ≤ IV; 4. MoCA attention dimension scores 2–5 points; 5. Kinesthetic and Visual Imagery Questionnaire score ≥ 55 points; 6. Ability to walk continuously for at least 10 m (with/without assistance); 7. Good comprehension and verbal communication skills	1. Moderate and severe cognitive impairment (MoCA<18); 2. Hemineglect; 3. Aphasia; 4. Intense lower-extremity pain or spasticity hindering pedaling (MAS > 2); 5. Other conditions causing cognitive/attention deficits; 6. Skull defects affecting electrode-skin fitting	BCI-LLR + VR	14/16	FMA-LE, TUG, BBS, SDMT	4 W
Wan C	2024	1. Within 1–12 months of stroke onset (ischemic infarct or intracerebral hemorrhage); 2. Age ≥18 years and ≤75 years; 3. Lower-extremity Brunnstrom stage ≤IV; 4. Montreal Cognitive Assessment (MoCA) attention dimension scores of 2–5 points; 5. Score ≥55 on the Kinesthetic and Visual Imagery Questionnaire; 6. Ability to walk continuously for at least 10 meters with or without assistance; 7. Good comprehension and communication skills to participate and cooperate effectively	1. Moderate and severe cognitive impairment (MoCA <18); 2. Hemineglect (meet left lateralized neglect diagnostic criteria by the Chinese Test of Behavioral Neglect-Hong Kong version); 3. Aphasia (meet the aphasia diagnostic criteria by the China Rehabilitation Research Center Aphasia Examination); 4. Inability to focus on and comprehend screen information; 5. Intense pain of the lower-extremity or spasticity that hinders pedaling training; 6. Presence of other conditions leading to cognitive impairment or attention deficits (e.g., Alzheimer disease, Parkinson disease); 7. Skull defects affecting electrode-skin fitting	BCI-LLR + VR	9	FMA-LE, TUG, BBS, SDMT, MoCA	4 W
He J	2025	1. Age 35–79 years; 2. First subcortical ischemic stroke onset 2 weeks to 3 months; 3. Hemiplegia, proximal upper limb strength 1–3; 4. Right-handed; 5. Sitting balance ≥1; 6. Able to cooperate	1. MMSE<20; 2. Severe pain or limited limb mobility	BCI (MI + MA) + pedaling robot (upper & lower limbs)	25/23	FMA-UE, FMA-LE, EMG, EEG (DAR/DABR), fNIRS	2 W

### Results of individual studies

#### Lower limb function

All four included studies reported improvements in lower limb function following BCI-LLR training, though the magnitude and statistical significance varied across outcome measures.

Fugl-Meyer Assessment for Lower Extremity (FMA-LE). Yuan (Yuan et al., 2021) found that the BCI group showed a mean increase of 4.5 points compared to 2.1 points in the control group, with a significant between-group difference (*p* = 0.022). Similarly, the single-arm study by Wan et al. (2025a) reported a significant improvement of 4.78 points (*p* = 0.002). In contrast, the RCT by Wan et al. (2025b) observed no significant between-group difference (*p* = 0.476), although both groups improved significantly within groups (BCI: +4.36, *p* < 0.01; control: +3.63, *p* < 0.01). He et al. (2025) also found no significant between-group difference in FMA-LE (*p* = 0.392), with both groups showing improvement.

Timed Up and Go Test (TUG). Two studies reported TUG outcomes. Wan et al. (2025b) demonstrated that the BCI group had a significantly greater reduction in TUG time compared to the control group (−12.03 s vs. −4.65 s, between-group *p* = 0.038). The single-arm study by Wan et al. (2025a) similarly reported a significant reduction of 12.02 s (*p* = 0.008).

Berg Balance Scale (BBS). Two studies reported BBS outcomes. Wan et al. (2025b) found that the BCI group showed a significantly greater improvement in BBS scores compared to the control group (+5.50 vs. +3.38, between-group *p* = 0.042). Wan et al. (2025a) also reported a significant improvement of 4.00 points (*p* = 0.009).

Details of lower limb function outcomes for each study are presented in Table 3.

**Table 3:** Lower limb function outcomes.

Study	Outcome	Group	Baseline	Post-intervention	Change
Yuan et al. (2021)	FMA-L (0–34)	BCI	12.5 (IQR 8.8–20.0)	17.00	+4.5*
		Control	13.5 (IQR 8.2–19.0)	15.60	+2.1
Wan et al. (2025b)	FMA-LE (0–34)	BCI	17.29 ± 5.25	21.64 ± 4.50	+4.36**
		Control	20.50 ± 6.63	24.00 ± 5.15	+3.63**
	TUG (s)	BCI	52.29 ± 19.41	40.27 ± 16.10	−12.03**†
		Control	40.06 ± 27.74	34.15 ± 21.87	−4.65*
	BBS (0–56)	BCI	31.43 ± 12.79	36.93 ± 12.09	+5.50**†
		Control	37.13 ± 9.82	40.50 ± 8.73	+3.38**
Wan et al. (2025a)	FMA-LE	BCI (single-arm)	17.00 ± 5.98	21.78	+4.78**
	TUG (s)	BCI	58.30 ± 30.99	46.28	−12.02**
	BBS	BCI	33.00 ± 13.69	37.00	+4.00**
He et al. (2025)	FMA-LE (0–34)	BCI	18.0 (IQR 11.0–26.0)	24.0 (IQR 18.0–29.0)	+6
		Control	14.0 (IQR 11.0–20.5)	23.0 (IQR 18.5–28.0)	+9

Values are mean ± SD or median (IQR) as originally reported. Change values are mean change except He et al. (2025) (median change).

*p < 0.05, **p < 0.01. ^†^Between-group difference significant.

#### Cognitive function

Symbol Digit Modalities Test (SDMT). Three studies assessed SDMT. Wan et al. (2025b) observed that the BCI group had a significantly greater improvement than the control group (+5.29 vs. +3.19, between-group *p* = 0.013). Yuan similarly found a significant between-group difference favoring the BCI group (*p* = 0.036). The single-arm study by Wan et al. (2025a) reported a significant improvement of 3.22 points (*p* = 0.047).

Montreal Cognitive Assessment (MoCA). The single-arm study by Wan et al. (2025a) reported a significant improvement of 1.89 points (*p* = 0.044). However, the RCT by Yuan found no significant change in MoCA scores (*p* = 0.672).

Attention Index. All three studies that measured the attention index (based on beta/alpha EEG power ratio) reported a significant increasing trend over the training period. Yuan reported *p* = 0.002; Wan et al. (2025b) reported a mean increase of 7.02 (*p* = 0.005); Wan et al. (2025a) reported an increase of approximately 7 points (*p* = 0.005).

Cognitive function outcomes are summarized in Table 4.

**Table 4:** Cognitive function outcomes.

Study	Outcome	Group	Baseline	Post-intervention	Change
Yuan et al. (2021)	MoCA (0–30)	BCI	15.0 (IQR 9.8–21.0)	–	+0.2
		Control	11.5 (IQR 8.2–18.0)	–	+0.2
	SDMT	BCI	7.0 (IQR 3.8–17.2)	–	+1.1*†
		Control	7.0 (IQR 3.0–12.5)	–	0
	Attention index	BCI	–	–	+**
Wan et al. (2025b)	SDMT	BCI	18.29 ± 9.97	23.57 ± 9.26	+5.29**†
		Control	22.00 ± 11.40	25.19 ± 11.36	+3.19**
	Attention index	BCI	–	–	+7.02**
	MoCA	–	–	–	–
Wan et al. (2025a)	MoCA	BCI (single-arm)	25.56 ± 3.57	27.45	+1.89*
	SDMT	BCI	19.00 ± 10.71	22.22	+3.22*
	Attention index	BCI	–	–	+7**
	TMT-A (s)	BCI	141.56 ± 71.94	–	No change

Values are mean ± SD or median (IQR) as originally reported.

*p < 0.05, **p < 0.01. ^†^Between-group difference significant. “–” not reported.

#### Neurophysiological measures

Only He reported neurophysiological outcomes. The BCI group demonstrated significant decreases in DAR (*p* = 0.009) and DABR (*p* < 0.001) compared to baseline and to the control group, indicating improved brain states. EMG analysis revealed significant increases in deltoid and biceps muscle activity in the BCI group (*p* < 0.01). fNIRS showed enhanced functional connectivity in the prefrontal cortex, supplementary motor area, and primary motor cortex in the BCI group, suggesting neuroplastic changes.

Neurophysiological measures are summarized in Table 5.

**Table 5:** Neurophysiological measures.

Study	Measure	Group	Baseline	Post-intervention
He et al. (2025)	DAR	BCI	Higher	Decreased**†
		Control	Higher	No change
	DABR	BCI	Higher	Decreased**†
		Control	Higher	No change
	Deltoid RMS (shoulder)	BCI	Low	Increased**
	Biceps RMS (elbow)	BCI	Low	Increased**
	Control EMG	Control	–	No significant change
	fNIRS connectivity	BCI	–	Increased* (PFC-SMA-M1)
		Control	–	Limited changes

*p < 0.05, **p < 0.01. ^†^Between-group difference significant. “–” not reported.

## Discussion

This systematic review included 4 studies with a total of 117 patients. The rehabilitation effect of BCI-LLR was analyzed from two aspects: lower limb movement and cognition. The results suggest that BCI-LLR may improve FMA, SDMT and Attention Index. Evidence for MoCA was inconsistent, with only one single-arm study showing improvement and one RCT finding no significant change. TUG and BBS showed significant improvement in one RCT and one single-arm study, but further validation is needed. Possible explanations include: (1) insufficient statistical power due to the tiny sample size; (2) the short intervention periods (2–4 weeks) may be inadequate to produce measurable changes in these domains.

### Lower limb function

FMA is a comprehensive score for the reflex activity, joint activity, coordination ability and speed of the lower limbs (Zhang et al., 2024). The FMA evaluates in detail the quality of motor function recovery in patients after stroke. It is currently the most commonly used stroke motor function assessment scale in the world. BBS comprehensively evaluates static and dynamic balance functions (Lee et al., 2022). TUG quickly evaluates functional mobility, dynamic balance and fall risk (Park et al., 2021). The combination of the three can comprehensively reflect the patient’s motor function status.

Our results suggest that BCI-LLR may improve FMA. BCI-LLR improves patient neuroplasticity and functional recovery by completing high-intensity, repetitive gait training tasks and allowing patients to experience vestibular and subjective sensory stimulation during training (Wu et al., 2023). Previous meta-analyses of conventional lower-limb robots (without BCI) have reported similar effect sizes for FMA improvement (Hao et al., 2025). This suggests that BCI-LLR may offer comparable benefits, though head-to-head comparisons are lacking. It has been hypothesized that BCI-LLR might enhance neuroplasticity through motor imagery and real-time feedback. However, our limited data cannot confirm this mechanism. Future studies should incorporate neuroimaging or electrophysiological measures to explore the underlying neural mechanisms.

Previous meta-analyses of conventional lower-limb robots (without BCI) reported a pooled FMA MD of 3.74 (95% CI: 3.02–4.46, *p* < 0.05) (Huang et al., 2024). The observed improvements in FMA-LE across the included studies ranged from 2.1 to 4.8 points. Direct comparison across studies is inappropriate given differences in patient populations and intervention protocols; therefore, head-to-head RCTs are required.

### Cognitive function

MoCA is a more sensitive and comprehensive cognitive function screening tool than MMSE (Mini Mental State Examination) (Pinto et al., 2019; Suda et al., 2020). SDMT is a special in-depth assessment that specifically evaluates information processing speed, continuous attention and visual scanning capabilities (Vishwanath et al., 2024). In the cognitive assessment process, MoCA is often used first for overall screening, and then SDMT is used to focus on assessing the areas with the most obvious damage.

Our results suggest that BCI-LLR may be effective for improving SDMT. This suggests that brain-computer-based BCI-LLR may have an impact on cognitive recovery. The Attention Index is calculated based on the ratio of beta-band and alpha-band signals in the forehead EEG. Researchers often use the energy ratio in the beta and alpha bands as a quantitative measure of attention level. 3 papers found that the average attention index (beta and alphased band power ratio) of participants in the BCI-LLR group showed a fluctuating and increasing trend, suggesting an improvement in attentional focus.

He et al. (2025) reported that BCI-LLR training significantly enhanced functional connectivity between the prefrontal cortex, supplementary motor area, and primary motor cortex, while reducing delta/alpha and delta/(alpha+beta) power ratios. These neuroplastic changes, absent in the control group, suggest that BCI-LLR promotes cortical reorganization through top-down network reinforcement. Thus, multimodal neurophysiological evidence supports a neural basis for BCI-LLR-induced motor recovery.

The observed improvements in FMA-LE across studies ranged from 2.1 to 4.8 points, which is below the commonly cited minimal clinically important difference (MCID) of 6 points (Mishra et al., 2024). For BBS, the MCID varies depending on patient stage and assessment method: distribution-based estimates range from 2.3 to 4.9 points, while anchor-based estimates range from 6.5–12.5 points for acute phase patients and 13.5 points for chronic phase patients (Mishra et al., 2024). The BBS improvements observed in this review(range 4.0–5.5 points) fall at the lower bound of the MCID range, leaving their clinical significance uncertain. Furthermore, the MCID for SDMT in stroke patients has not been fully established in the literature. Therefore, although statistically significant improvements were observed across various indicators, the clinical importance of these findings has not been firmly established.

Strokes cause lasting disability by disrupting the connectivity of the brain’s functional network (Karnadipa et al., 2025). Based on our findings, BCI-LLR may promote motor recovery by restoring functional brain networks. However, the number of studies included in this analysis is limited, and the actual clinical significance is difficult to determine, and a large number of clinical trials are needed for verification.

## Conclusion

Current evidence from a limited number of pilot studies suggests that BCI-LLR may have potential benefits for lower limb motor function, information processing speed and attention index in stroke patients. However, due to the critically limited sample size and the lack of evidence for effects on global cognition and mobility, the efficacy of BCI-LLR remains unconfirmed. Large-scale, well-powered randomized controlled trials are urgently needed before any clinical recommendations can be made. Future research should also directly compare BCI-LLR with conventional robotic rehabilitation.

### Limitations

1. This is a relatively new intervention method that has not been verified by a large number of experiments. Because of this, this systematic review included only a small number of studies, which may affect the robustness and generalizability of the findings.
2. Only the effectiveness between BCI-LLR and traditional lower limb training was compared, and the differences between BCI-LLR and ordinary lower limb training robots were not compared.
3. Because the included trials required in-person patient participation, blinding of the intervention was almost impossible. Three of the four included trials lacked blinding of participants and personnel, leading to high risk of performance bias. Only one trial (He et al., 2025) successfully implemented blinding using a sham feedback control.

### Strengths

1. Previous studies have analyzed the effect on the upper limb. This paper analyzes the effect of BCI-LLR on the lower limb.
2. For the first time, this paper includes the improvement of cognition by lower-limb robots into the discussion.
3. A novel concept proposed in this paper is to rehabilitate brain networks involved in cognition as a whole, rather than focusing on the lower limbs alone. It provides a new direction for the further development of lower limb rehabilitation after stroke.

## Data Availability

The original contributions presented in the study are included in the article/supplementary material, further inquiries can be directed to the corresponding authors.

## References

[ref1] Baniqued P. D. E. Stanyer E. C. Awais M. Alazmani A. Jackson A. E. Mon-Williams M. A. . (2021). Brain–computer interface robotics for hand rehabilitation after stroke: a systematic review. J. Neuroeng. Rehabil. 18:15. doi: 10.1186/s12984-021-00820-8, 33485365 PMC7825186

[ref2] Elameer M. Lumley H. Moore S. A. Marshall K. Alton A. Smith F. E. . (2023). A prospective study of MRI biomarkers in the brain and lower limb muscles for prediction of lower limb motor recovery following stroke. Front. Neurol. 14:1229681. doi: 10.3389/fneur.2023.1229681, 37941576 PMC10628497

[ref3] Esposito D. Centracchio J. Andreozzi E. Gargiulo G. D. Naik G. R. Bifulco P. (2021). Biosignal-based human–machine interfaces for assistance and rehabilitation: a survey. Sensors 21:6863. doi: 10.3390/s21206863, 34696076 PMC8540117

[ref4] Hao Q.-H. Qiu M.-M. Wang J. Tu Y. Lv Z. H. Zhu T. M. (2025). The effect of lower limb rehabilitation robot on lower limb-motor function in stroke patients: a systematic review and meta-analysis. Syst. Rev. 14:70. doi: 10.1186/s13643-025-02759-6, 40140968 PMC11938605

[ref5] He J. Yuan Z. Quan L. Xi H. Guo J. Zhu D. . (2025). Multimodal assessment of a BCI system for stroke rehabilitation integrating motor imagery and motor attempts: a randomized controlled trial. J. Neuroeng. Rehabil. 22:185. doi: 10.1186/s12984-025-01723-8, 40859358 PMC12379318

[ref6] Hornby T. G. Reisman D. S. Ward I. G. Scheets P. L. Miller A. Haddad D. . (2020). Clinical practice guideline to improve locomotor function following chronic stroke, incomplete spinal cord injury, and brain injury. J. Neurol. Phys. Ther. 44, 49–100. doi: 10.1097/NPT.0000000000000303, 31834165

[ref7] Hossain K. M. Islam M. A. Hossain S. Nijholt A. Ahad M. A. R. (2023). Status of deep learning for EEG-based brain–computer interface applications. Front. Comput. Neurosci. 16:1006763. doi: 10.3389/fncom.2022.1006763, 36726556 PMC9885375

[ref8] Hu M.-M. Wang S. Wu C.-Q. Li K. P. Geng Z. H. Xu G. H. . (2024). Efficacy of robot-assisted gait training on lower extremity function in subacute stroke patients: a systematic review and meta-analysis. J. Neuroeng. Rehabil. 21:165. doi: 10.1186/s12984-024-01463-1, 39300491 PMC11411785

[ref9] Huang H. Su X. Zheng B. Cao M. Zhang Y. Chen J. (2024). Effect and optimal exercise prescription of robot-assisted gait training on lower extremity motor function in stroke patients: a network meta-analysis. Neurol. Sci. 46, 1151–1167. doi: 10.1007/s10072-024-07780-6, 39312061

[ref10] Karnadipa T. Chong B. Malik M. Shim V. Fernandez J. Stinear C. . (2025). Motor network efficiency in stroke patients: comparing activation likelihood estimation-based and whole brain parcellations. Annu. Int. Conf. IEEE Eng. Med. Biol. Soc 2025, 1–4. doi: 10.1109/EMBC58623.2025.11253164, 41335664

[ref11] Khalid S. Malik A. N. Siddiqi F. A. Rathore F. A. (2023). Overview of gait rehabilitation in stroke. J. Pak. Med. Assoc. 73, 1142–1145. doi: 10.47391/JPMA.23-39 37218257

[ref12] Le Franc S. Herrera Altamira G. Guillen M. Butet S. Fleck S. Lécuyer A. . (2022). Toward an adapted neurofeedback for post-stroke motor rehabilitation: state of the art and perspectives. Front. Hum. Neurosci. 16:917909. doi: 10.3389/fnhum.2022.917909, 35911589 PMC9332194

[ref13] Lee J. Chun M. H. Seo Y. J. Lee A. Choi J. Son C. (2022). Effects of a lower limb rehabilitation robot with various training modes in patients with stroke: a randomized controlled trial. Medicine 101:e31590. doi: 10.1097/MD.0000000000031590, 36343085 PMC9646640

[ref14] Li M. Li H. Yu H. (2024). Research status of lower limb exoskeleton rehabilitation robot. Sheng Wu Yi Xue Gong Cheng Xue Za Zhi 41, 833–839. doi: 10.7507/1001-5515.202211055, 39218611 PMC11366457

[ref15] Li D.-X. Zha F.-B. Long J.-J. Liu F. Cao J. Wang Y. L. (2021). Effect of robot assisted gait training on motor and walking function in patients with subacute stroke: a random controlled study. J. Stroke Cerebrovasc. Dis. 30:105807. doi: 10.1016/j.jstrokecerebrovasdis.2021.105807, 33895428

[ref16] Liu X. Zhang W. Li W. Zhang S. Lv P. Yin Y. (2023). Effects of motor imagery based brain-computer interface on upper limb function and attention in stroke patients with hemiplegia: a randomized controlled trial. BMC Neurol. 23:136. doi: 10.1186/s12883-023-03150-5, 37003976 PMC10064693

[ref17] Mishra B. Sudheer P. Agarwal A. Nilima N. Srivastava M. V. P. Vishnu V. Y. (2024). Minimal clinically important difference of scales reported in stroke trials: a review. Brain Sci. 14:80. doi: 10.3390/brainsci14010080, 38248295 PMC10813687

[ref18] Momtazmanesh S. Moghaddam S. S. Ghamari S.-H., and GBD 2019 Chronic Respiratory Diseases Collaborators (1990). Global burden of chronic respiratory diseases and risk factors, 1990–2019: an update from the global burden of disease study 2019. EClinicalMedicine 59:101936. doi: 10.1016/j.eclinm.2023.101936, 37229504 PMC7614570

[ref19] Park C. Son H. Yeo B. (2021). The effects of lower extremity cross-training on gait and balance in stroke patients: a double-blinded randomized controlled trial. Eur. J. Phys. Rehabil. Med. 57, 4–12. doi: 10.23736/s1973-9087.20.06183-3, 32891079

[ref20] Pinto T. C. C. Machado L. Bulgacov T. M. Rodrigues-Júnior A. L. Costa M. L. G. Ximenes R. C. C. . (2019). Is the montreal cognitive assessment (MoCA) screening superior to the mini-mental state examination (MMSE) in the detection of mild cognitive impairment (MCI) and Alzheimer’s disease (AD) in the elderly? Int. Psychogeriatr. 31, 491–504. doi: 10.1017/S1041610218001370, 30426911

[ref21] Purrahman D. Shojaeian A. Poniatowski Ł. A. Piechowski-Jóźwiak B. Mahmoudian-Sani M. R. (2023). The role of progranulin (PGRN) in the pathogenesis of ischemic stroke. Cell. Mol. Neurobiol. 43, 3435–3447. doi: 10.1007/s10571-023-01396-8, 37561339 PMC11410000

[ref22] Saini V. Guada L. Yavagal D. R. (2021). Global epidemiology of stroke and access to acute ischemic stroke interventions. Neurology 97, S6–S16. doi: 10.1212/wnl.0000000000012781, 34785599

[ref23] Suda S. Muraga K. Ishiwata A. Nishimura T. Aoki J. Kanamaru T. . (2020). Early cognitive assessment following acute stroke: feasibility and comparison between mini-mental state examination and montreal cognitive assessment. J. Stroke Cerebrovasc. Dis. 29:104688. doi: 10.1016/j.jstrokecerebrovasdis.2020.104688, 32063455

[ref24] Vishwanath S. Hopper I. Cloud G. C. Chong T. T. J. Shah R. C. Donnan G. A. . (2024). The impact of incident stroke on cognitive trajectories in later life. Alzheimer’s Res. Ther. 16:111. doi: 10.1186/s13195-024-01479-8, 38762556 PMC11102228

[ref25] Wan C. Zhang W. Nie Y. Qian Y. Wang J. Xu H. . (2025a). Impact of motor imagery-based brain-computer interface combined with virtual reality on enhancing attention, executive function, and lower-limb function in stroke: a pilot study. PM R. 17, 811–821. doi: 10.1002/pmrj.13324, 39992067

[ref26] Wan C. Zhang Q. Qiu Y. Zhang W. Nie Y. Zeng S. . (2025b). Effects of dual-task mode brain-computer interface based on motor imagery and virtual reality on balance and attention in patients with stroke: a randomized controlled pilot trial. J. Neuroeng. Rehabil. 22:187. doi: 10.1186/s12984-025-01730-9, 40883792 PMC12395916

[ref27] Wu J. Liu Y. Zhao J. Jia Z. (2023). Research on a new rehabilitation robot for balance disorders. IEEE Trans. Neural Syst. Rehabil. Eng. 31, 3927–3936. doi: 10.1109/TNSRE.2023.3312692, 37676800

[ref28] Yuan Z. Peng Y. Wang L. Song S. Chen S. Yang L. . (2021). Effect of BCI-controlled pedaling training system with multiple modalities of feedback on motor and cognitive function rehabilitation of early subacute stroke patients. IEEE Trans. Neural Syst. Rehabil. Eng. 29, 2569–2577. doi: 10.1109/TNSRE.2021.3132944, 34871175

[ref29] Zhang Y. Zhao W. Wan C. Wu X. Huang J. Wang X. . (2024). Exoskeleton rehabilitation robot training for balance and lower limb function in sub-acute stroke patients: a pilot, randomized controlled trial. J. Neuroeng. Rehabil. 21:98. doi: 10.1186/s12984-024-01391-0, 38851703 PMC11162020

[ref30] Żukowska Z. Krawczyk M. Poniatowski Ł. A. (2025). Alterations in supine position mobility and dynamics in post-stroke individuals with hemiparesis compared to neurologically intact controls: a video-based observational assessement. J. Clin. Med. 14:7949. doi: 10.3390/jcm14227949, 41302985 PMC12653150

